# Comparison of surgery rates in biologic-naïve patients with Crohn’s disease treated with vedolizumab or ustekinumab: findings from SOJOURN

**DOI:** 10.1186/s12876-023-02723-5

**Published:** 2023-03-25

**Authors:** Michelle Vu, Sabyasachi Ghosh, Kandavadivu Umashankar, Laura Weber, Christina Landis, Ninfa Candela, Benjamin Chastek

**Affiliations:** 1 Optum, Eden Prairie, MN USA; 2 Takeda Pharmaceuticals U.S.A., Inc, Lexington, MA USA

**Keywords:** Crohn’s disease, Inflammatory bowel disease, Biologic, Vedolizumab, Ustekinumab, Surgery

## Abstract

**Background:**

Crohn’s disease (CD) is a chronic inflammatory bowel disease characterized by relapsing and remitting inflammation that leads to progressive bowel damage. Despite advances in medical treatment for CD, many patients require surgical intervention. Most studies of surgery rates are from patients treated with anti-tumor necrosis factor alpha (anti-TNFα) treatments, with comparatively little data on the surgery rates of patients treated with vedolizumab and ustekinumab. SOJOURN aimed to estimate the hazard rate and incidence of the first CD-related surgery following initiation of treatment with vedolizumab or ustekinumab in biologic-naïve patients with CD.

**Methods:**

SOJOURN was a retrospective, observational cohort study examining administrative claims data from the Optum® Research Database between July 1, 2017 and March 31, 2020. Included participants were adults with a diagnosis of CD and a claim for vedolizumab or ustekinumab (defined as the index treatment) between January 1, 2018 and December 31, 2019, with no claims for a biologic in the 6 months before initiation of this treatment. The variable follow-up started on the day after the index date and continued until whichever came first of discontinuation of the index treatment, surgery event, switching of the index treatment, initiation of combination biologic treatment, disenrollment, or March 31, 2020. The time to the first CD-related surgery on biologic treatment was estimated by Kaplan–Meier analysis. The hazard ratio and incidence rate ratio of CD-related surgery for each treatment cohort was compared using a Cox proportional hazards model and a Poisson regression model, respectively.

**Results:**

Of the 1,122 included patients, 578 received vedolizumab and 544 received ustekinumab. After 1 year of the variable follow-up, 7.7% of patients receiving vedolizumab and 11.6% of patients receiving ustekinumab had undergone a CD-related surgery. Vedolizumab was associated with a 34.2% lower hazard rate of surgery (hazard ratio 0.658, 95% confidence interval [CI] 0.436–0.994, *p* = 0.047) and a 34.5% lower incidence of surgery (rate ratio 0.655, 95% CI 0.434–0.988, *p* = 0.044) than ustekinumab.

**Conclusions:**

This real-world analysis of biologic-naïve patients with CD suggests that vedolizumab is associated with greater effectiveness in reducing the rate of CD-related surgery than ustekinumab.

**Supplementary Information:**

The online version contains supplementary material available at 10.1186/s12876-023-02723-5.

## Background

Crohn’s disease (CD) is a chronic inflammatory bowel disease (IBD). The condition is characterized by skip lesions and transmural inflammation that can affect any region of the gastrointestinal tract [1, 2]. CD has a relapsing and remitting course with periods of inflammation causing progressive bowel damage over time, resulting in complications such as strictures, fistulas, and abscesses [3].

Although the majority of patients have uncomplicated disease at diagnosis, approximately 34% of patients develop stricturing or penetrating complications within 5 years [4]. Medical treatment for moderate to severe CD includes conventional therapies (such as 5-aminosalicylates, corticosteroids, and immunomodulators) and advanced therapies such as biologics. Biologics include anti-tumor necrosis factor alpha (anti-TNFα) treatments, as well as ustekinumab (an antagonist of interleukin-12/23) and vedolizumab (an anti-α4β7-integrin) [5]. Although biologics have significantly improved patient outcomes [6], patients with an inadequate response or nonadherence to medical treatments and those who develop disease complications may still require surgery [7]. Surgery rates have declined in recent years, owing in part to advances in medical treatment [8, 9]; however, the cumulative risk of first major abdominal surgery (intestinal resection) within 10 years of diagnosis remains as high as 26.2% [10]. Surgery for CD is associated with a risk of complications, is noncurative, and incurs significant costs to the healthcare system [11, 12]. Therefore, there is value in understanding the role of biologics in reducing surgery rates.

Most surgery rate data are from patients treated with an anti-TNFα treatment [13–16], which have been available for more than two decades [6]. The newer biologics ustekinumab and vedolizumab have demonstrated efficacy over placebo for the induction and maintenance of remission [17–19], but little is known about their impact on surgery rates. Furthermore, no head-to-head clinical trials have compared the efficacy of vedolizumab and ustekinumab. There is only limited real-world evidence on the comparative effectiveness of these biologics and data are only available from patients with CD for whom anti-TNFα treatment has failed [20–22].

In this article, we report the real-world surgery rates in biologic-naïve patients receiving vedolizumab or ustekinumab.

## Methods

### Objectives

The primary objective of SOJOURN (Surgery rate cOmparison between treatment JOURNey with ustekinumab and vedolizumab in biologic-naïve Crohn’s disease patients) was to compare the hazard rate and the incidence of the first CD-related surgery following initiation of treatment with vedolizumab or ustekinumab in patients with CD who were previously biologic-naïve. An exploratory objective was to compare the proportion of patients in the vedolizumab and ustekinumab treatment groups who underwent each type of CD-related surgery.

### Study design

**Fig. 1:**
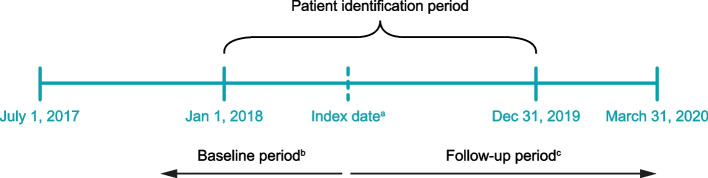
Study design. ^a^The index date was defined as the first medical or pharmacy claim for vedolizumab or ustekinumab during the patient identification period. ^b^The baseline period was defined as the period 6 months before and on the index date. ^c^The follow-up period began on the day after the index date and ended on whichever came first of discontinuation, surgery event, switching or initiation of combination treatment, disenrollment, or the end of the study period

SOJOURN was a retrospective, observational cohort study conducted using medical and pharmacy claims data from the Optum® Research Database between July 1, 2017 and March 31, 2020 (Fig. 1).

### Data source

The Optum® Research Database is a de-identified and Health Insurance Portability and Accountability Act compliant database that includes medical and pharmacy claims data from 1993 onwards for more than 73 million lives in the USA. Socio-economic characteristics are also linked to the administrative claims data.

### Study population

Patients with a new medical or pharmacy claim for either vedolizumab or ustekinumab during the identification period (January 1, 2018 to December 31, 2019) were included, with the date of the first claim defined as the index date. The baseline period was defined as the 6 months before the index date. In order to minimize the number of patients lost to follow-up, the study design used a variable follow-up dependent on the length of time a patient received the index treatment and a surgery event. This variable follow-up period began on the day after the index date and ended on whichever came first of discontinuation of the index treatment, surgery event, switching of the index treatment, initiation of combination biologic treatment, disenrollment, or the end of the study period (March 31, 2020). Eligible patients were required to have an International Classification of Diseases, tenth revision (ICD-10-CM) diagnosis code for CD (Table S1) in any position during the baseline or follow-up period, be at least 18 years old during the year of the index date, and to be continuously enrolled in a healthcare plan with medical and pharmacy benefits during the baseline period and for at least 1 month after the index date. Patients were excluded for: medical or pharmacy claims for vedolizumab, ustekinumab, or other biologics or advanced therapies approved for IBD (adalimumab, infliximab [and biosimilars], certolizumab, natalizumab, golimumab, or tofacitinib) during the baseline period; a diagnosis of rheumatoid arthritis, ankylosing spondylitis, psoriatic arthritis, plaque psoriasis, hidradenitis suppurativa, or noninfectious uveitis during the baseline or follow-up period; or a greater number of claims with a diagnosis code of ulcerative colitis than for CD during the follow-up period (Table S1).

### Baseline variables

Demographics were ascertained from data recorded on the index date or the first available claim after the index date. Demographics included age, index year, sex, race/ethnicity, US region, and insurance type. Clinical characteristics were ascertained from claims during the baseline period and included Charlson Comorbidity Index score, CD-related surgery, disease location, CD characteristics (including the presence of perianal disease, abscess, and fistulizing and stricturing disease), comorbid conditions of depression or anxiety, and the use of corticosteroids and other nonbiologic medications. Healthcare resource utilization (both CD-related and all-cause utilization) was ascertained from claims during the baseline period, and included ambulatory visits, emergency room visits, inpatient stays, and pharmacy use. Healthcare resource utilization was categorized as CD-related if the claim included an ICD-10-CM diagnosis code for CD in any position or if the claim was for the index treatment.

### Endpoints

The primary endpoint was CD-related surgery during the variable follow-up period, identified from medical claims data using relevant Current Procedural Terminology (CPT), Healthcare Common Procedure Coding System (HCPCS), and ICD surgery procedure codes. For surgeries in the inpatient hospital setting, the admission date of the inpatient stay was captured. For surgeries in a non-inpatient setting, the date of the claim with a surgery procedure code was used. The time to surgery was defined as the number of days from the start of follow-up to the first occurrence of CD-related surgery. The incidence of CD-related surgery during the variable follow-up period was calculated using the number of patients who underwent a CD-related surgery during follow-up as the numerator and the patient follow-up time as the denominator.

The occurrence of CD-related surgery by surgery type was an exploratory endpoint. CD-related surgeries were classified by surgery type if the medical claims contained either a CPT procedure code for the surgery type or an ICD-10-CM procedure code and a diagnosis code for CD. CD-related surgery categories included abscess drainage, abscess drainage with fistula repair, seton placement, fistula repair or stricturoplasty, and excision or resection of the small or large intestine.

### Statistical analysis

Baseline demographics and clinical characteristics were analyzed using descriptive statistics for the overall cohort and stratified by treatment group. Bivariate comparisons were conducted using the appropriate test (t-test, Wilcoxon rank-sum test, or chi-squared test) based on the distribution of each variable, using a significance level of α = 0.05 for a two-tailed test.

To account for the variable length of follow-up, incidence was reported as the number of patients with surgery per patient-month. Kaplan–Meier analysis was used to estimate the time to occurrence of CD-related surgery for each treatment group, followed by a log rank test to determine whether the distributions for the treatment groups were different.

A Cox proportional hazards model was used to determine whether the risk of first CD-related surgery differed between the treatment groups after controlling for covariates. Time at risk was defined by censoring at the date of discontinuation of the index treatment, switching of the index treatment, initiation of combination treatment with another biologic, disenrollment, or the end of the study period. A Poisson regression model was used to determine whether CD-related surgery incidences differed between treatment groups after controlling for covariates. CD-related surgery types were calculated as counts and proportions of patients in each treatment group. Analyses controlled for the following covariates at baseline: sex, age, insurance type, race/ethnicity, Charlson Comorbidity Index score, abscess, anemia, corticosteroid use, and CD-related hospitalization.

All statistical analyses were conducted using SAS v9.4, SAS Institute Inc., Cary, NC.

## Results

### Patient attrition

**Fig. 2:**
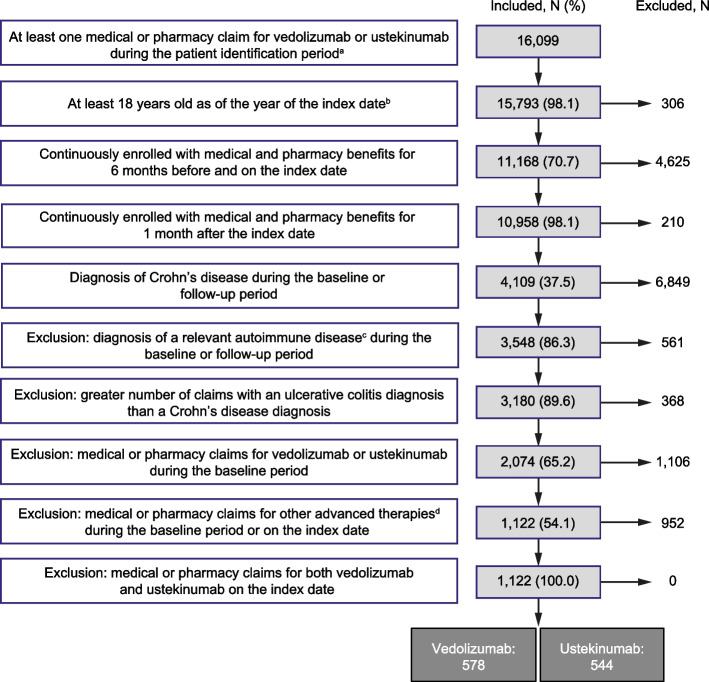
Patient attrition. ^a^January 1, 2018 to December 31, 2019. ^b^The index date was defined as the first medical or pharmacy claim for vedolizumab or ustekinumab during the patient identification period. ^c^Rheumatoid arthritis, ankylosing spondylitis, psoriatic arthritis, plaque psoriasis, hidradenitis suppurativa, or noninfectious uveitis.^d^Other advanced therapies approved for the treatment of Crohn’s disease or ulcerative colitis

After applying the eligibility criteria, the study cohort included 1,122 biologic-naïve patients with CD, of whom 578 and 544 were included in the vedolizumab and ustekinumab treatment groups, respectively (Fig. 2).

### Baseline demographics, clinical characteristics, and healthcare resource utilization

The baseline demographics and clinical characteristics of the study cohort are shown in Table S2.

The median age of patients in the overall cohort was 47 years, with a greater proportion of women (54.7%) than men (Table S2). Most patients were White (78.5%) and located in the South (40.0%) or the Midwest (31.7%) of the USA. Most patients (74.2%) were commercially insured. Patients in the vedolizumab group were on average older (mean age, 49.8 vs. 45.8 years, *p* < 0.001) and had a greater mean Charlson Comorbidity Index score than patients who received ustekinumab (0.76 vs. 0.53, *p* = 0.004). A greater proportion of patients treated with vedolizumab were insured by Medicare than patients in the ustekinumab group (32.0 vs. 19.3%, *p* < 0.001).

Fewer than one-tenth of patients (8.3%) in the overall cohort had undergone CD-related surgery at baseline. When stratified by treatment group, a numerically greater proportion of patients receiving ustekinumab (9.9%) had undergone CD-related surgery than those receiving vedolizumab (6.7%); however, this difference was not statistically significant (*p* = 0.05).

More than half of patients in the overall cohort had ileocolonic disease (55.3%). Disease location differed between the treatment groups, with a greater proportion of patients in the ustekinumab group having ileocolonic disease (59.0% vs. 51.9%, *p* = 0.02) and a lower proportion having colonic only disease (10.8% vs. 17.5%, *p* = 0.002) than those in the vedolizumab group. The proportion of patients who had ileal-only disease was similar between the treatment groups (vedolizumab 20.1%, ustekinumab 21.5%, *p* = 0.55). A greater proportion of patients receiving ustekinumab had perianal or severe rectal disease (8.5% vs. 5.4%, *p* = 0.04) and fistulas or fistulizing disease (19.9% vs. 12.8%, *p* = 0.001) than those receiving vedolizumab.

Approximately half of the patients (50.8%) had received corticosteroids for at least 14 days at baseline in the overall cohort, with no significant difference between the treatment groups. A greater proportion of patients in the vedolizumab group had received 5-aminosalicylates at baseline than those in the ustekinumab group (25.8% vs. 16.9%, *p* < 0.001).

Healthcare resource utilization, including CD-related utilization and all-cause utilization, was similar between the treatment groups; in the overall cohort, almost all patients had a CD-related ambulatory visit (98.3%), 20.9% had a CD-related emergency room visit, and 20.8% had a CD-related inpatient stay.

### Unadjusted analysis of Crohn’s disease-related surgery

During the variable follow-up period, 8.6% of patients in the overall cohort underwent CD-related surgery (Table 1). In total, 42 (7.3%) patients receiving vedolizumab underwent CD-related surgery compared with 54 (9.9%) patients receiving ustekinumab (*p* = 0.11).

**Table 1:** Proportion of patients who underwent Crohn’s disease-related surgery and time to first Crohn’s disease-related surgery

	**Total** ***N* = 1,122**	**Vedolizumab** ***N* = 578**	**Ustekinumab** ***N* = 544**	***p* value**
CD-related surgery, n (%)	96 (8.56)	42 (7.27)	54 (9.93)	0.111
Time from index date to first CD-related surgery, days				
Mean (SD)	207.17 (177.35)	229.86 (188.91)	189.52 (167.46)	0.271
Median (Q1–Q3)	158.00 (57.00–310.50)	170.00 (77.00–324.00)	141.50 (49.00–258.00)	

*CD* Crohn’s disease, *Q* Quartile, *SD* Standard deviation

**Fig. 3:**
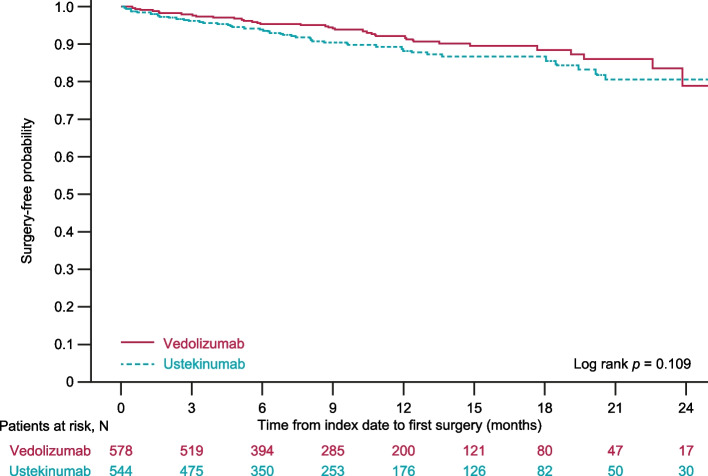
Kaplan–Meier curve for time to first Crohn’s disease-related surgery on biologic treatment

The mean time from the index date to the date of first CD-related surgery was 230 days for patients receiving vedolizumab and 190 days for patients receiving ustekinumab (*p* = 0.27, Table 1). The incidence of CD-related surgery was 0.0069 surgeries per patient-month for vedolizumab and 0.0093 surgeries per patient-month for ustekinumab (rate ratio 0.743, *p* = 0.15). At 1 year of follow-up, the cumulative incidence of CD-related surgery was 7.7% of patients in the vedolizumab group and 11.6% of the ustekinumab group; there was no statistically significant difference between the time to first surgery survival curves at 2 years after the index date (log rank *p* = 0.11, Fig. 3).

### Adjusted analysis of Crohn’s disease-related surgery

**Fig. 4:**
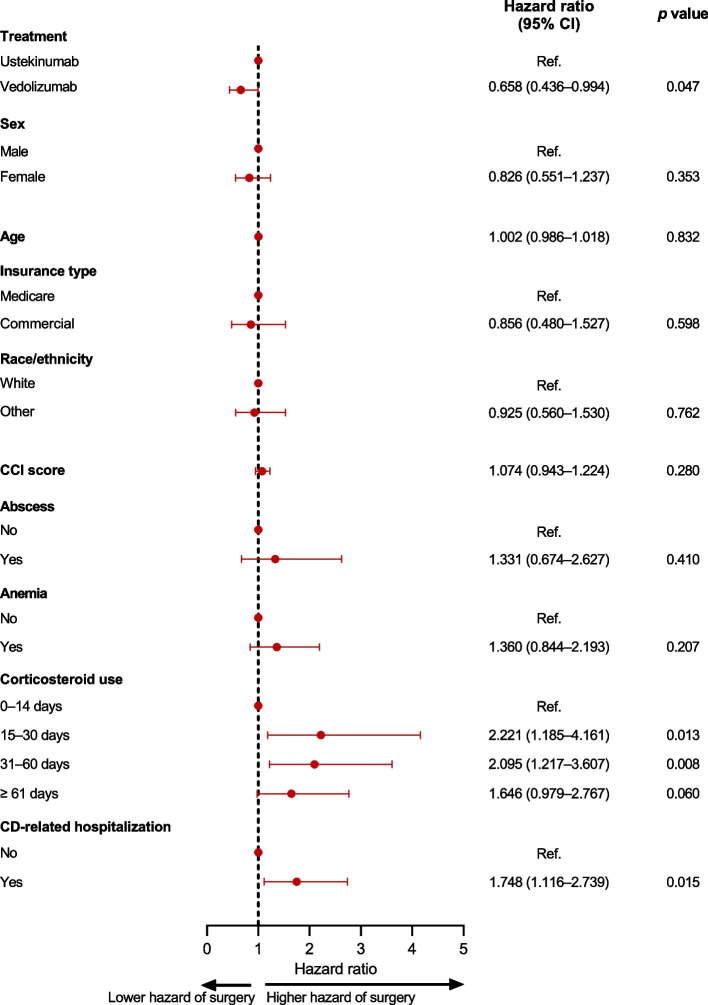
Adjusted hazard ratios for Crohn’s disease-related surgery. CCI, Charlson Comorbidity Index; CD, Crohn’s disease; CI, confidence interval

After adjusting for baseline demographics and clinical characteristics, vedolizumab was associated with a 34.2% lower hazard rate of CD-related surgery than ustekinumab (hazard ratio 0.658, 95% confidence interval [CI] 0.436–0.994, *p* = 0.047, Fig. 4).

**Fig. 5:**
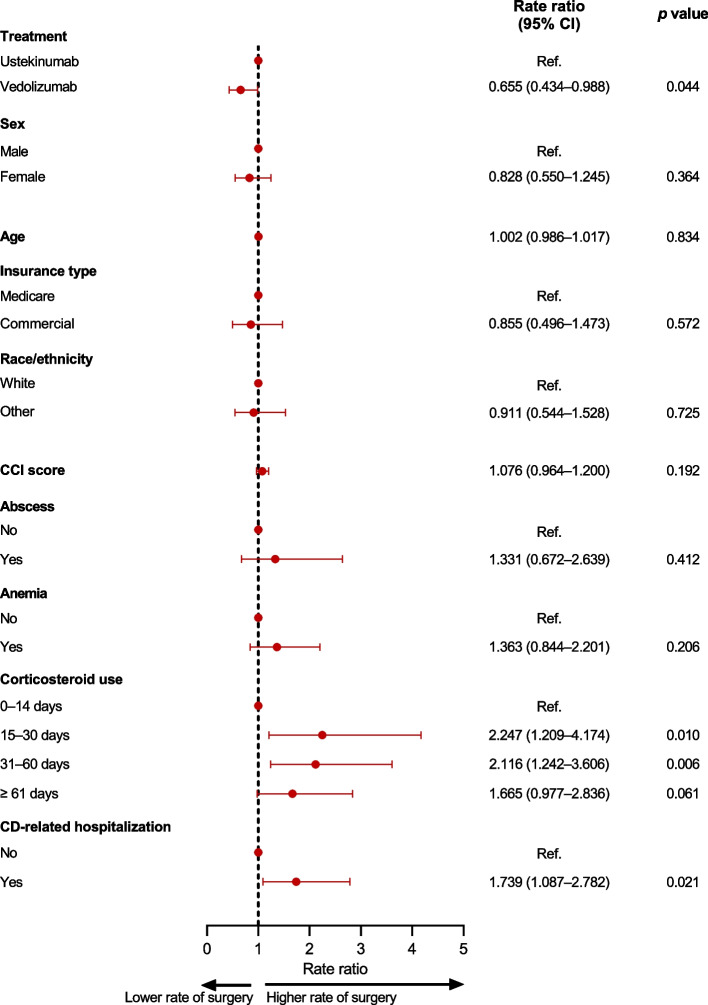
Adjusted rate ratios for Crohn’s disease-related surgery. CCI, Charlson Comorbidity Index; CD, Crohn’s disease; CI, confidence interval

The CD-related surgery rate was 34.5% lower among patients who received vedolizumab than those who received ustekinumab (rate ratio 0.655, 95% CI 0.434–0.988, *p* = 0.04, Fig. 5), after adjustments for baseline covariates.

Other than biologic treatment group, prior CD-related hospitalization and the use of corticosteroids during the baseline period (for 15–30 days and 31–60 days relative to fewer than 15 days) were associated with both a significantly greater hazard rate and significantly higher incidence of CD-related surgery (Figs. 4 and 5).

### Crohn’s disease-related surgery type and subtype

The CD-related surgery types are shown in Table 2. In the overall cohort, the most common CD-related surgery type was intestinal excision or resection (4.8%), followed by fistula repair or stricturoplasty (3.3%) and abscess drainage (2.0%).

**Table 2:** Proportion of patients who underwent each Crohn’s disease-related surgery type

CD-related surgery type, n (%)	Total *N* = 1,122	Vedolizumab *N* = 578	Ustekinumab *N* = 544	*p* value
Abscess drainage	22 (1.96)	13 (2.25)	9 (1.65)	0.473
Abscess drainage with fistula repair	4 (0.36)	1 (0.17)	3 (0.55)	0.288
Seton placement	12 (1.07)	4 (0.69)	8 (1.47)	0.205
Fistula repair/stricturoplasty	37 (3.30)	15 (2.60)	22 (4.04)	0.174
Excision/resection: overall	54 (4.81)	22 (3.81)	32 (5.88)	0.104
Excision/resection of the small or large intestine^a^	1 (0.09)	0 (0.00)	1 (0.18)	0.302
Excision/resection of the large intestine	47 (4.19)	20 (3.46)	27 (4.96)	0.209
Excision/resection of the small intestine	33 (2.94)	10 (1.73)	23 (4.23)	0.013

A surgery event was attributed to a category if it contained at least one of the procedure codes corresponding to the surgery category type

*CD* Crohn’s disease

^a^The surgery procedure code did not specify the location

The proportions of patients who underwent each CD-related surgery type and subtype were similar between patients in the vedolizumab and ustekinumab groups (Table 2). The only notable difference observed was the proportion of patients who underwent excision or resection of the small intestine, which was significantly higher for those in the ustekinumab group than the vedolizumab group (4.2% vs. 1.7%, *p* = 0.01).

## Discussion

Despite marked reductions in the patient-level risk of surgery over the past two decades in people with CD, the 5-year cumulative risk of surgery for patients diagnosed with CD since the year 2000 remains about 18% [10]. Patients with CD incur higher costs to the healthcare system than patients without IBD, with CD-related surgery accounting for the highest direct costs [11]. Additionally, CD-related surgery carries a risk of post-surgery complications [12]. It is therefore important to understand the impact of biologics on reducing surgery rates in patients with CD. Previous studies have reported surgery rate data for patients treated with anti-TNFα treatments [13–16], but there is a lack of data on surgery rates for other biologics, such as vedolizumab and ustekinumab.

This real-world, observational cohort study aimed to compare the rates of CD-related surgery in patients treated with vedolizumab or ustekinumab. The overall surgery rate at 1 year was less than 10%; a lower rate than has been reported previously for patients with CD [10]. The unadjusted analyses indicated that a numerically lower proportion of patients treated with vedolizumab underwent CD-related surgery and the mean time to the first CD-related surgery was numerically longer compared to ustekinumab; however, these unadjusted comparisons were not statistically significant. However, after adjustment for baseline demographics and disease characteristics, vedolizumab was associated with a 34% lower hazard rate and a 35% lower incidence of CD-related surgery compared with ustekinumab.

Previous real-world studies have indicated that ustekinumab may have superior effectiveness to vedolizumab in patients with CD [20–22]. However, these studies, which focused on clinical response and remission rates, were restricted to small cohorts of patients who were refractory to anti-TNFα treatment [20–22], whereas the present study specifically evaluated the comparative effectiveness of ustekinumab and vedolizumab in reducing CD-related surgeries in relatively large cohorts of patients who were biologic-naïve. CD-related surgery could be considered a proxy for disease progression since the complications that often necessitate surgical intervention are more likely to develop over time with progressive bowel damage [3, 23]. Therefore, these findings suggest that vedolizumab may be more effective than ustekinumab in reducing disease progression when used as a first-line treatment in biologic-naïve patients.

Aside from treatment group, previous CD-related hospitalization and 15–60 days of corticosteroid use at baseline were also associated with a greater hazard rate and incidence of CD-related surgery in the models. Patients may be hospitalized for CD owing to exacerbation of inflammation or disease complications [24], which are also indications for surgery [7]. Corticosteroids are recommended for induction of remission in the short term [25, 26]; therefore, their use at baseline may also be indicative of a disease flare or inadequately controlled disease, which may result in surgery. Although disease severity was not investigated in this study, this association between surgery risk and CD-related hospitalization or corticosteroid use at baseline may indicate that baseline disease severity is also associated with an increased risk of CD-related surgery.

The CD-related surgery types and subtypes were generally similar between treatment groups; however, excision or resection of the small intestine was more common in the ustekinumab group than the vedolizumab group. This difference is likely explained by the differences in disease location at baseline between the treatment groups. Although the proportion of patients with ileal only disease was similar in each treatment group, a greater proportion of patients receiving ustekinumab had ileocolonic disease than those receiving vedolizumab, and a smaller proportion of patients receiving ustekinumab had colonic-only disease.

To our knowledge, SOJOURN is the first study to evaluate the real-world comparative effectiveness of vedolizumab or ustekinumab in biologic-naïve patients with CD. The Optum® Research Database includes claims data for patients enrolled in a commercial or Medicare Advantage health plan. The database is national in scope and includes a range of geographic regions and employer groups. Furthermore, the models used to interrogate the endpoint of CD-related surgery demonstrated very similar results, despite having different assumptions. The Poisson model assumes that the risk of CD-related surgery is constant during follow-up, whereas the Cox proportional hazards model assumes that the ratio between the two groups is constant. Further strengthening these findings, the models were adjusted for several demographic and clinical characteristics that could otherwise confound the results.

As SOJOURN is a retrospective study conducted using administrative claims data, the results should be interpreted in the context of the following limitations. First, the study cohort was considered to be biologic-naïve based on the absence of claims for a biologic in the 6-month baseline period; however, patients who received a biologic before this time may have been included. Second, patient eligibility, variables, and outcomes were based on the presence of diagnosis, treatment, or procedure codes and administrative claims data, which may be inaccurate or missing. Moreover, a pharmacy claim for prescription of a particular drug does not guarantee patient compliance with the recommended dosing regimen. Indeed, insurance and shipment delays may result in unused, but claimed for, doses of medication. Third, patients treated with vedolizumab may have more opportunity to report CD-related complications than patients treated with ustekinumab because vedolizumab is administered intravenously in a medical setting, whereas ustekinumab is self-administered by patients subcutaneously. However, despite this surveillance bias for potentially detecting more surgeries in patients treated with vedolizumab, there were fewer CD-related surgeries reported for the vedolizumab cohort than the ustekinumab cohort. Fourth, in order to minimize the number of patients lost to follow-up, SOJOURN used a variable follow-up period with a median of 9.3 months (range, 0.4–27.2 months), and therefore could not estimate the 5- and 10-year risk of CD-related surgery. However, the surgery rates reported at 1 year were similar to, or lower than, that reported previously [10], with an overall surgery rate in the present study of less than 10%. At 2 years of follow-up, the limited sample size available likely contributed to the convergence of the Kaplan–Meier curve at this timepoint. Finally, disease activity, duration, severity, and the presence of biomarkers at baseline may impact effectiveness outcomes. These data are not available in administrative claims databases and therefore could not be accounted for in this study. Baseline characteristics such as the presence of complications, CD-related hospitalization, disease location, and corticosteroid use were captured, which may be considered as proxies of disease severity. However, there may be other differences in disease history or risk factors between the treatment populations that are unable to be accounted for from the available claims data. Any such differences, if present, could be a potential source of bias.

## Conclusions

In conclusion, the results of this real-world study suggest that vedolizumab is associated with a lower rate of CD-related surgery than ustekinumab. While these claims data should be interpreted with potential sources of bias in mind, they provide the first insight into the comparative outcomes of vedolizumab and ustekinumab for CD when administered as first-line biologics. These results may help to inform clinicians’ choice and positioning of biologics.

## Supplementary Information

**Additional file 1:** **TableS1.** Diagnostic codes for inclusion and exclusion criteria. **TableS2.** Baseline demographics and clinical characteristics.

## Data Availability

The data that support the findings of this study are available from Optum but restrictions apply to the availability of these data, which were used under license for the current study, and so are not publicly available. Data are however available from the corresponding author upon reasonable request and with permission of Optum.
