# Supplementary material for: Comparison of surgery rates in biologic-naïve patients with Crohn’s disease treated with vedolizumab or ustekinumab: findings from SOJOURN
Source: BMC Gastroenterol. 2023 Mar 25;23:87. doi: 10.1186/s12876-023-02723-5 (PMC10039499; doi:10.1186/s12876-023-02723-5)
Supplement: Supplementary file 1 — Additional file 1: TableS1. Diagnostic codes for inclusion and exclusion criteria. TableS2. Baseline demographics and clinical characteristics. [file 12876_2023_2723_MOESM1_ESM.docx]

**Supplemental Information**

**Table S1 Diagnostic codes for inclusion and exclusion criteria**

| **Diagnosis** | **ICD-10-CM code** |
| --- | --- |
| *Inclusion* |  |
| Crohn’s disease | K5000, K50011, K50012, K50013, K50014, K50018, K50019, K5010, K50111, K50112, K50113, K50114, K50118, K50119, K5080, K50811, K50812, K50813, K50814, K50818, K50819, K5090, K50911, K50912, K50913, K50914, K50918, K50919 |
| *Exclusion* |  |
| Ulcerative colitis | K5100, K51011, K51012, K51013, K51014, K51018, K51019, K5120, K51211, K51212, K51213, K51214, K51218, K51219, K5130, K51311, K51312, K51313, K51314, K51318, K51319, K5140, K51411, K51412, K51413, K51414, K51418, K51419, K5150, K51511, K51512, K51513, K51514, K51518, K51519, K5180, K51811, K51812, K51813, K51814, K51818, K51819, K5190, K51911, K51912, K51913, K51914, K51918, K51919 |
| Rheumatoid arthritis | M0500, M05011, M05012, M05019, M05021, M05022, M05029, M05031, M05032, M05039, M05041, M05042, M05049, M05051, M05052, M05059, M05061, M05062, M05069, M05071, M05072, M05079, M0509, M0510, M05111, M05112, M05119, M05121, M05122, M05129, M05131, M05132, M05139, M05141, M05142, M05149, M05151, M05152, M05159, M05161, M05162, M05169, M05171, M05172, M05179, M0519, M0520, M05211, M05212, M05219, M05221, M05222, M05229, M05231, M05232, M05239, M05241, M05242, M05249, M05251, M05252, M05259, M05261, M05262, M05269, M05271, M05272, M05279, M0529, M0530, M05311, M05312, M05319, M05321, M05322, M05329, M05331, M05332, M05339, M05341, M05342, M05349, M05351, M05352, M05359, M05361, M05362, M05369, M05371, M05372, M05379, M0539, M0540, M05411, M05412, M05419, M05421, M05422, M05429, M05431, M05432, M05439, M05441, M05442, M05449, M05451, M05452, M05459, M05461, M05462, M05469, M05471, M05472, M05479, M0549, M0550, M05511, M05512, M05519, M05521, M05522, M05529, M05531, M05532, M05539, M05541, M05542, M05549, M05551, M05552, M05559, M05561, M05562, M05569, M05571, M05572, M05579, M0559, M0560, M05611, M05612, M05619, M05621, M05622, M05629, M05631, M05632, M05639, M05641, M05642, M05649, M05651, M05652, M05659, M05661, M05662, M05669, M05671, M05672, M05679, M0569, M0570, M05711, M05712, M05719, M05721, M05722, M05729, M05731, M05732, M05739, M05741, M05742, M05749, M05751, M05752, M05759, M05761, M05762, M05769, M05771, M05772, M05779, M0579, M057A, M0580, M05811, M05812, M05819, M05821, M05822, M05829, M05831, M05832, M05839, M05841, M05842, M05849, M05851, M05852, M05859, M05861, M05862, M05869, M05871, M05872, M05879, M0589, M058A, M059, M0600, M06011, M06012, M06019, M06021, M06022, M06029, M06031, M06032, M06039, M06041, M06042, M06049, M06051, M06052, M06059, M06061, M06062, M06069, M06071, M06072, M06079, M0608, M0609, M060A, M061, M0620, M06211, M06212, M06219, M06221, M06222, M06229, M06231, M06232, M06239, M06241, M06242, M06249, M06251, M06252, M06259, M06261, M06262, M06269, M06271, M06272, M06279, M0628, M0629, M0630, M06311, M06312, M06319, M06321, M06322, M06329, M06331, M06332, M06339, M06341, M06342, M06349, M06351, M06352, M06359, M06361, M06362, M06369, M06371, M06372, M06379, M0638, M0639, M064, M0680, M06811, M06812, M06819, M06821, M06822, M06829, M06831, M06832, M06839, M06841, M06842, M06849, M06851, M06852, M06859, M06861, M06862, M06869, M06871, M06872, M06879, M0688, M0689, M068A, M069, M0800, M08011, M08012, M08019, M08021, M08022, M08029, M08031, M08032, M08039, M08041, M08042, M08049, M08051, M08052, M08059, M08061, M08062, M08069, M08071, M08072, M08079, M0808, M0809, M080A, M0820, M08211, M08212, M08219, M08221, M08222, M08229, M08231, M08232, M08239, M08241, M08242, M08249, M08251, M08252, M08259, M08261, M08262, M08269, M08271, M08272, M08279, M0828, M0829, M082A, M083, M0840, M08411, M08412, M08419, M08421, M08422, M08429, M08431, M08432, M08439, M08441, M08442, M08449, M08451, M08452, M08459, M08461, M08462, M08469, M08471, M08472, M08479, M0848, M084A |
| Ankylosing spondylitis | M081, M450, M451, M452, M453, M454, M455, M456, M457, M458, M459 |
| Psoriatic arthritis | L4050, L4051, L4052, L4053, L4054, L4059 |
| Plaque psoriasis | L400 |
| Hidradenitis suppurativa | L732 |
| Noninfectious uveitis | D8683, H2000, H20011, H20012, H20013, H20019, H20021, H20022, H20023, H20029, H20041, H20042, H20043, H20049, H2010, H2011, H2012, H2013, H2020, H2021, H2022, H2023, H20811, H20812, H20813, H20819, H209, H3020, H3021, H3022, H3023, H44111, H44112, H44113, H44119, H44131, H44132, H44133, H44139 |

ICD-10-CM, International Classification of Diseases, tenth revision.

**Table S2 Baseline demographics and clinical characteristics**

|  | Total  N = 1122 | Vedolizumab  N = 578 | Ustekinumab  N = 544 | *p* value |
| --- | --- | --- | --- | --- |
| Demographics | | | | |
| Age, years | | | |  |
| Mean (SD) | 47.83 (16.70) | 49.78 (17.69) | 45.76 (15.33) | < 0.001 |
| Median (Q1–Q3) | 47.00 (34.00–61.00) | 49.00 (35.00–66.00) | 45.00 (33.00–58.00) |  |
| Age group, years, n (%) | | | | < 0.001 |
| 18–34 | 290 (25.85) | 140 (24.22) | 150 (27.57) | 0.200 |
| 35–49 | 333 (29.68) | 156 (26.99) | 177 (32.54) | 0.042 |
| 50–64 | 278 (24.78) | 127 (21.97) | 151 (27.76) | 0.025 |
| ≥ 65 | 221 (19.70) | 155 (26.82) | 66 (12.13) | < 0.001 |
| Index year, n (%) | | | | 0.055 |
| 2018 | 493 (43.94) | 238 (41.18) | 255 (46.88) |  |
| 2019 | 629 (56.06) | 340 (58.82) | 289 (53.13) |  |
| Sex, n (%) | | | | 0.971 |
| Male | 508 (45.28) | 262 (45.33) | 246 (45.22) |  |
| Female | 614 (54.72) | 316 (54.67) | 298 (54.78) |  |
| Race/ethnicity, n (%) | | | | 0.702 |
| White | 881 (78.52) | 449 (77.68) | 432 (79.41) | 0.481 |
| African American/Black | 91 (8.11) | 49 (8.48) | 42 (7.72) | 0.643 |
| Asian | 27 (2.41) | 17 (2.94) | 10 (1.84) | 0.228 |
| Hispanic | 67 (5.97) | 35 (6.06) | 32 (5.88) | 0.903 |
| Unknown/uncoded | 22 (1.96) | 9 (1.56) | 13 (2.39) | 0.315 |
| No socioeconomic status information | 34 (3.03) | 19 (3.29) | 15 (2.76) | 0.605 |
| US region, n (%) | | | | 0.565 |
| Northeast | 132 (11.76) | 71 (12.28) | 61 (11.21) | 0.578 |
| Midwest | 356 (31.73) | 190 (32.87) | 166 (30.51) | 0.397 |
| South | 449 (40.02) | 228 (39.45) | 221 (40.63) | 0.687 |
| West | 183 (16.31) | 87 (15.05) | 96 (17.65) | 0.240 |
| Other | 1 (0.09) | 1 (0.17) | 0 (0.00) | 0.332 |
| Unknown | 1 (0.09) | 1 (0.17) | 0 (0.00) | 0.332 |
| Insurance type, n (%) | | | | < 0.001 |
| Commercial | 832 (74.15) | 393 (67.99) | 439 (80.70) |  |
| Medicare | 290 (25.85) | 185 (32.01) | 105 (19.30) |  |
| Clinical characteristics | | | | |
| Baseline CCI score |  |  |  |  |
| Mean (SD) | 0.65 (1.33) | 0.76 (1.43) | 0.53 (1.19) | 0.004 |
| Median (Q1–Q3) | 0.00 (0.00–1.00) | 0.00 (0.00–1.00) | 0.00 (0.00–1.00) |  |
| Baseline CCI score category, n (%) | | | | 0.014 |
| 0 | 795 (70.86) | 390 (67.47) | 405 (74.45) | 0.010 |
| 1–2 | 244 (21.75) | 134 (23.18) | 110 (20.22) | 0.229 |
| 3–4 | 56 (4.99) | 39 (6.75) | 17 (3.13) | 0.005 |
| 5–10 | 27 (2.41) | 15 (2.60) | 12 (2.21) | 0.671 |
| ≥ 10 | 0 (0.00) | 0 (0.00) | 0 (0.00) | – |
| CD-related surgery, n (%) | 93 (8.29) | 39 (6.75) | 54 (9.93) | 0.054 |
| Abscess drainage | 21 (1.87) | 9 (1.56) | 12 (2.21) | 0.423 |
| Abscess drainage with fistula repair | 2 (0.18) | 1 (0.17) | 1 (0.18) | 0.966 |
| Seton placement | 14 (1.25) | 5 (0.87) | 9 (1.65) | 0.234 |
| Fistula repair/stricturoplasty | 26 (2.32) | 11 (1.90) | 15 (2.76) | 0.342 |
| Excision/resection – small or large intestine | 58 (5.17) | 27 (4.67) | 31 (5.70) | 0.437 |
| Excision/resection – large intestine – specific | 49 (4.37) | 22 (3.81) | 27 (4.96) | 0.343 |
| Excision/resection – small intestine – specific | 37 (3.30) | 17 (2.94) | 20 (3.68) | 0.491 |
| Disease location, n (%) | | | | 0.006 |
| Ileum–colon | 621 (55.35) | 300 (51.90) | 321 (59.01) | 0.017 |
| Ileum | 233 (20.77) | 116 (20.07) | 117 (21.51) | 0.553 |
| Colon | 160 (14.26) | 101 (17.47) | 59 (10.85) | 0.002 |
| Unspecified | 108 (9.63) | 61 (10.55) | 47 (8.64) | 0.277 |
| Disease characteristics, n (%) | | | |  |
| Perianal/severe rectal disease | 77 (6.86) | 31 (5.36) | 46 (8.46) | 0.041 |
| Abscess | 85 (7.58) | 40 (6.92) | 45 (8.27) | 0.392 |
| Fistula/fistulizing disease | 182 (16.22) | 74 (12.80) | 108 (19.85) | 0.001 |
| Stricture/stricturing disease | 3 (0.27) | 1 (0.17) | 2 (0.37) | 0.528 |
| Mental disorder, n (%) | 317 (28.25) | 174 (30.10) | 143 (26.29) | 0.156 |
| Depression | 189 (16.84) | 98 (16.96) | 91 (16.73) | 0.919 |
| Anxiety | 241 (21.48) | 127 (21.97) | 114 (20.96) | 0.679 |
| Anemia, n (%) | 214 (19.07) | 102 (17.65) | 112 (20.59) | 0.210 |
| Use of corticosteroids, n (%) |  |  |  |  |
| 0–14 days | 562 (50.09) | 275 (47.58) | 287 (52.76) | 0.083 |
| 15–30 days | 121 (10.78) | 61 (10.55) | 60 (11.03) | 0.797 |
| 31–60 days | 183 (16.31) | 105 (18.17) | 78 (14.34) | 0.083 |
| 61+ days | 256 (22.82) | 137 (23.70) | 119 (21.88) | 0.466 |
| Use of other nonbiologic medications, n (%) | 381 (33.96) | 216 (37.37) | 165 (30.33) | 0.013 |
| 5-aminosalicylates | 241 (21.48) | 149 (25.78) | 92 (16.91) | < 0.001 |
| Immunomodulators | 178 (15.86) | 91 (15.74) | 87 (15.99) | 0.909 |
| Healthcare resource utilization | | | | |
| CD-related utilization, n (%) |  |  |  |  |
| Ambulatory visit | 1103 (98.31) | 569 (98.44) | 534 (98.16) | 0.715 |
| Office visit | 1028 (91.62) | 521 (90.14) | 507 (93.20) | 0.064 |
| Outpatient visit | 880 (78.43) | 446 (77.16) | 434 (79.78) | 0.287 |
| Emergency room visit | 235 (20.94) | 124 (21.45) | 111 (20.40) | 0.666 |
| Inpatient stay | 233 (20.77) | 128 (22.15) | 105 (19.30) | 0.241 |
| Pharmacy use | 143 (12.75) | 27 (4.67) | 116 (21.32) | < 0.001 |
| All-cause utilization, n (%) |  |  |  |  |
| Ambulatory visit | 1119 (99.73) | 578 (100.00) | 541 (99.45) | 0.074 |
| Office visit | 1100 (98.04) | 566 (97.92) | 534 (98.16) | 0.774 |
| Outpatient visit | 1013 (90.29) | 524 (90.66) | 489 (89.89) | 0.664 |
| Emergency room visit | 432 (38.50) | 232 (40.14) | 200 (36.76) | 0.246 |
| Inpatient stay | 254 (22.64) | 142 (24.57) | 112 (20.59) | 0.111 |
| Pharmacy use | 1055 (94.03) | 541 (93.60) | 514 (94.49) | 0.531 |

CCI, Charlson Comorbidity Index; CD, Crohn’s disease; Q, quartile; SD, standard deviation.
